# Ellagic Acid Modulates the Amyloid Precursor Protein Gene via Superoxide Dismutase Regulation in the Entorhinal Cortex in an Experimental Alzheimer’s Model

**DOI:** 10.3390/cells10123511

**Published:** 2021-12-13

**Authors:** Wafaa S. Ramadan, Saleh Alkarim

**Affiliations:** 1 Department of Anatomy, Faculty of Medicine, King Abdulaziz University, Jeddah 21589, Saudi Arabia; 2 Department of Biological Sciences, Faculty of Science, King Abdulaziz University, Jeddah 21589, Saudi Arabia; skarim@kau.edu.sa

**Keywords:** Alzheimer’s, entorhinal cortex, ellagic acid, superoxide dismutase, caspase3, amyloid precursor protein

## Abstract

Patients suffering from Alzheimer’s disease (AD) are still increasing worldwide. The development of (AD) is related to oxidative stress and genetic factors. This study investigated the therapeutic effects of ellagic acid (EA) on the entorhinal cortex (ERC), which plays a major role in episodic memory, in the brains of an AD rat model. AD was induced using AlCl_3_ (50 mg/kg orally for 4 weeks). Rats were divided into four groups: control, AD model, EA (treated with 50 mg/kg EA orally for 4 weeks), and ADEA (AD rats treated with EA after AlCl_3_ was stopped) groups. All rats were investigated for episodic memory using the novel object recognition test (NORT), antioxidant serum biomarkers, lipid peroxidation, histopathology of the ERC, and quantitative PCR for the superoxide dismutase (*SOD*) gene. EA therapy in AD rats significantly increased the discrimination index for NORT and the levels of SOD, glutathione, and total antioxidant capacity. Lipid peroxidation products were decreased, and the neurofibrillary tangles and neuritic plaques in the ERC sections were reduced after EA administration. The decrease in ERC thickness in the AD group, caused by caspase-3-mediated apoptosis and neurotoxicity due to amyloid precursor protein, was modulated by the increased *SOD* mRNA expression. Adjustment of the ERC antioxidant environment and decreased oxidative stress under EA administration enhanced SOD expression, resulting in the modulation of amyloid precursor protein toxicity and caspase-3-mediated apoptosis, thereby restoring episodic memory.

## 1. Introduction

Dementia causes a continuous decline in cognitive function and is associated with an increased burden on patients’ families and on society [1]. Alzheimer’s disease (AD), a common cause of dementia [2], is a neurodegenerative disorder that leads to impaired memory, a decline in mental functions, behavioral problems, and neuropsychiatric manifestations [3]. A World Health Organization report (2001) predicted a three-fold increase in the number of AD cases in the next 20 years, with a 125% increase in the Middle East and North Africa by 2050 [4]. 

AD is correlated with the autosomal dominant or sporadic inheritance of genes associated with amyloid precursor protein (APP), apolipoprotein E, and presenilin 1 and 2 [5]. An increase in APP levels has been associated with AD neurodegenerative changes and plaque formation in the brain. Excessive precipitation of amyloid plaques and microglia activity are associated with deterioration of cognition [6]. 

Neurodegenerative disorders have also been linked to oxidative stress and reactive oxygen species (ROS), which cause a decline in mitochondrial defense by altering Ca^2+^ homeostasis and membrane permeability, with the consequent release of cytochrome C and apoptosis [7]. Enhancing ROS provokes mitochondrial dysfunction which has an injurious effect on the cellular DNA, proteins, and lipids. Deposited Aβ precipitates oxidative stress, thus increasing ROS levels [8,9]. Hence, the increased level of ROS leads to release of proapoptotic signaling protein, CytC which enhances the formation of apoptosome, resulting in neurodegenerative disease [10] Moreover, the extracellular deposits of Aβ can attack mitochondria, resulting in altered mitochondrial membrane potential, resulting in loss of adenosine triphosphate (ATP) and increase ROS, leading to cell apoptosis [11,12]. 

Moreover, ROS and neuronal oxidation stimulate the signaling route that changes APP or tau processing [13]. Among various antioxidants, superoxide dismutase (SOD) plays a major role in the protection against ROS-induced neurodegeneration, plaque-dependent neuritic dystrophy, and APP(hAPP)/Aβ-induced impairment of the hippocampus and neocortex [14]. 

The interconnection between the entorhinal cortex (ERC) and hippocampus plays a pivotal role in episodic memory, and stores spatiotemporal information of past events. The ERC forms the prime connection between hippocampal formation and the neocortex [15]. The entorhinal cortex was found to be a common focus of pathology and the most affected cortical area in AD [16]. The early memory loss in AD is thought to be related to the progressive deterioration of the ERC and the targets of these pathways in the dentate gyrus and CA3 and CA1 areas in the hippocampus [17]. The hypothesis that AD originates in the ERC and spreads to other cortical and subcortical areas has been reinforced in both humans and rodents, and continues to be the predominant model in AD [18].

Despite the availability of several treatment options, AD progression is not easily controllable. Therefore, herbs and natural extracts are often used in the management of AD [19,20]. Their bioactive compounds, such as ellagitannins, have gained medical attention for their antioxidant, antiproliferative, and anticarcinogenic properties [21]. Ellagic acid, mostly present as ellagitannins, is available in various medicinal plants and fruits. During digestion ellagitannins are converted to ellagic acid which, when acted upon by gut microbiota, is transformed to a more active and bioavailable substance, urolithins (dibenzo[b,d]pyran-6-one derivatives) [22]. Lately, the pharmacological properties of EA on CNS became the focus for research, since it revealed a potential protective effect on many neurodegenerative diseases mainly due to its antioxidant and anti-inflammatory activity [23]. The intrinsic antioxidant capacity of EA was attributed to its radical scavenging activity and inhibition of lipid peroxidation properties [24]. Moreover, EA proved to hinder the pro-oxidative activity of metals as nickel and ferrous ion by chelation and reduced oxidative DNA damage [25]. 

The present study aimed to determine how the antioxidant EA modulates *SOD* and *APP* genes to alleviate the pathological features of AD. The ERC was investigated in this study because it is the main interconnection between the hippocampus and neocortex.

## 2. Materials and Methods

In total, 55 adult male Wistar rats (200–250 g) were purchased from the Faculty of Science, King Abdulaziz University, Jeddah, Saudi Arabia. The animals were housed in wire-mesh cages (5 animals/cage) under standard conditions of humidity, light/darkness cycle, and temperature in accordance with the Principles of Laboratory Animal Care. They were provided food and water ad libitum. 

All materials, including aluminum chloride (AlCl_3_) and EA, were purchased from Sigma-Aldrich (St. Louis, MO, USA).

### 2.1. Experimental Design

The rats were randomly divided into four groups: Group I (control; n = 5) received no medication; Group II (EA; n = 10) received EA (50 mg/kg), homogenized in water, orally for 4 weeks; Group III (AD; n = 20) received AlCl_3_ (50 mg/kg), dissolved in water, orally for 4 weeks to induce the AD model. This dose was chosen following previous studies which revealed reduction in cognitive function with least mortality of rats administering the same dose [26], and Group IV (ADEA; n = 20) received AlCl_3_ (50 mg/kg) orally for 4 weeks, followed by EA (50 mg/kg/day) orally for 2 weeks. EA (50 mg/kg) was approved as the lowest dose to restore the antioxidant defense system [27,28]. Oral medications were administered through an intragastric tube. 

### 2.2. Novel Object Recognition Test (NORT)

For the NORT, a white, cubic box (60 × 60 × 60 cm^3^) was used as the open field arena. Three identical, cylindrical, blue objects were used (O1, O2, and O3). One orange, square object was used as the novel object (N). A video camera was installed on top of the apparatus and was connected to EthoVision video tracking software (version xt8; Noldus Information Technology, Wageningen, The Netherlands) [29]. 

The test was performed in three stages: habituation, familiarization, and test stages. The duration of each stage was 10 min, separated by 6 h intervals. The entire procedure was performed on all rats in the four groups.

In the habituation stage, the animals were allowed to explore the empty box. In the familiarization stage, two objects (O1 and O2) were placed at two poles of the box, about 5 cm from the walls. The animals were allowed to explore them for 10 min. The procedure was repeated in the test stage, but O1 and O2 were replaced with O3 and N. Exploration was considered if the rats touched or sniffed the object with the nose [30]. Rats with normal memory functions explored N more. The box was cleaned between each stage.

The total exploration time in the test stage was calculated as the total time consumed exploring both objects using the discrimination index (DI) [31]:

$$DI=\frac{\left(N-O3\right)\times 100}{\left(N+O3\right)}$$

### 2.3. Antioxidant Biomarkers 

At the end of 6 weeks, blood was drawn from the tail veins and allowed to clot. Serum was prepared via centrifugation at 3000 rpm for 15 min and stored at −20 °C for biochemical analysis. The Activity Colorimetric Assay Kit (R&D Systems, Minneapolis, MN, USA) was used for the analysis of SOD, glutathione (GSH), and total antioxidant capacity (TAC) (Abcam, Cambridge, UK), following the manufacturers’ directions. For SOD, serum was diluted 1:5 with sample buffer. SOD samples and standard were laid out in duplicate in the well plate. Reaction was initiated by adding 20 µL of xanthine oxidase. Plates incubated on a shaker for 30 min. Absorbance read at 440–460 nm using a plate reader. One unit was defined as the amount of enzyme needed to exhibit fifty percent dismutation of the superoxide radical. SOD activity (U/mL) was standardized using Cytochrome C and xanthine oxidase coupled assay. 

For GSH, 160 µL of the Reaction Mix (NADPH Generating Mix and Glutathione Reaction Buffer) was incubated in each well plate for 10 min to generate NADPH before adding 20 µL of the GSH standard or sample solution. Then, 20 µL of substrate solution was added. Absorbance read at 405–415 nm using a microplate reader. The concentrations of GSH in the sample solutions using the standard glutathione calibration curve. 

For estimating TAC, an amount of 40 µL of sample dilution buffer was mixed with 10 µL of sample and incubated for 30 min at 37 °C. Then the wash solution was aspirated and the washing was repeated 5 times. Horseradish peroxidase (HRP)-conjugated reagent (50 µL) was added to each well except the blank control well and incubated for 30 min at 37 °C. Chromogen solution A (50 µL) and chromogen solution B (50 µL) were added to each well and mixed with gentle shaking and incubated at 37 °C for 15 min. Stop solution (50 µL) was added to each well to terminate the reaction. The samples were read at 450 nm using a microtiter plate reader.

### 2.4. Lipid Peroxidation

Thiobarbituric acid reactive substances (TBRS) were measured using the Activity Colorimetric Assay Kit (R&D Systems Inc., MN, Canada) according to the manufacturer’s instructions. Acid-treated samples and standards, followed by the 2-thiobarbituric acid (TBA) reagent, were added to the included 96-well microplate. The microplate was then incubated at 45–50 °C for 2–3 h, during which time the MDA in the sample reacts with the TBA reagent to produce a colored end product. The microplate was read at 532 nm and the intensity of the color corresponds to the level of lipid peroxidation in the sample.

### 2.5. Histopathological Study and Tissue Processing

After the completion of the behavioral experiments, sodium pentobarbital was intraperitoneally administered to anesthetize the rats. The rats were perfused with heparinized 0.9% NaCl by intracardiac infusion and then with 4% paraformaldehyde (pH = 7.4). After decapitation, their brains were extracted and half of the right cerebral hemispheres were incubated in sucrose (20%) for 3 days at 4 °C, and then frozen at −40 °C. Coronal sections (50 μm) were cut using a cryostat (at −19 °C) and silver stained to reveal neuritic plaques (NPs) and neurofibrillary tangles (NFTs) [32]. The remaining right cerebral hemispheres were kept in phosphate-buffered formalin for further tissue processing into paraffin blocks. Sections measuring 4 μm in thickness were stained with hematoxylin and eosin (H&E). The ERCs dissected from the left cerebral hemispheres were stored in RNAlater^®^ RNA Stabilization Solution for quantitative polymerase chain reaction (q-PCR) and preserved at −80 °C for further processing.

### 2.6. ERC Thickness

Coronal sections of the brain measuring 50 μm in thickness were prepared. The ERC was identified using a low magnification (×10). Measurements (mm) were performed on seven sections cut at regular intervals (every fifth section). For every section, a series of eight overlapping images was captured using an Olympus light microscope (BX51TF; Olympus, Tokyo, Japan). The entire thickness of the ERC was measured. The total ERC was outlined using Image-Pro Plus software (version 7.0.1; Media Cybernetics Inc., Rockville, MD, USA) in every image following the criteria in previous research [33]. The mean total thickness for each animal was calculated.

### 2.7. Immunohistochemistry

Brain sections cut from paraffin blocks were immunostained with anti-APP (A8717; Sigma-Aldrich) and anti-caspase 3 (06-735; Sigma-Aldrich). APP- and caspase 3-positive cells per visual field in the ERC were counted in five nonoverlapping visual fields in five sections for each animal using the Image-Pro Plus software (version 7.0.1; Media Cybernetics Inc.).

### 2.8. q-PCR

ERCs dissected from the left cerebral hemispheres which were stored in RNAlater^®^ RNA Stabilization Solution (Qiagen, Hilden, Germany) at −80 °C, were homogenized using a TissueLyser LT (Qiagen) in 1.0 mL TRIzol^®^ Reagent (Invitrogen Life Technologies, Paisley, UK) and total RNA was extracted according to standard procedures. The total extracted RNA was reverse transcribed into cDNA using the QuantiTect Reverse Transcription kit (QuantiTect^®^; Qiagen, Hilden, Germany, # cat no.205311) according to the manufacturer’s instructions using Thermo Hybaid PCR express (Thermo Scientific, Waltham, MA, USA).

The resultant cDNA was used to perform RT-qPCR by the QuantiTect SYBR-Green PCR kit (Qiagen, Hilden, Germany# cat no.204143) and the SOD Qiagen Quantitect Primer Assay (Rn_SOD_1_SG QuantiTect Primer assay, QT00174888) according to the manufacturer’s instructions. The reaction was run on the ABI 7500 Real-Time PCR system (Applied Biosystems; Thermo Fisher Scientific, Inc., Rockford, IL, USA). qPCR was conducted as follows: an initial polymerase activation at 95 °C for 10 min, then the samples were subjected to 40 cycles of denaturation at 95 °C for 15 s, 55 °C for 30 s, and 72 °C for 30 s in addition to a melting curve analysis at 60–95 °C. The 2^−∆∆Ct^ technique was used to measure the expression of the *SOD1* gene using Applied Biosystems 7500 software v2.3 (Applied Biosystems; Thermo Fisher Scientific, Inc., IL, USA). Reference gene (Hs_*GAPDH_1*_SG QuantiTect Primer assay, QT00079247) was used as an internal control to normalize the raw data of the samples and compare these results to a reference sample (Table 1). In this study, appropriate standardization strategies were carried out according to MIQE guidelines [34].

### 2.9. Statistical Analysis

The SPSS Statistics software version 20 (IBM Corp., Armonk, NY, USA) was used for data analysis. One-way analysis of variance (ANOVA) was used, and the least significant difference (LSD) *t*-test was employed when equal variance could be assumed. Data are presented as the means ± standard deviation (SD). Results were considered statistically significant at *p* < 0.05.

## 3. Results

### 3.1. NORT

**Figure 1:**
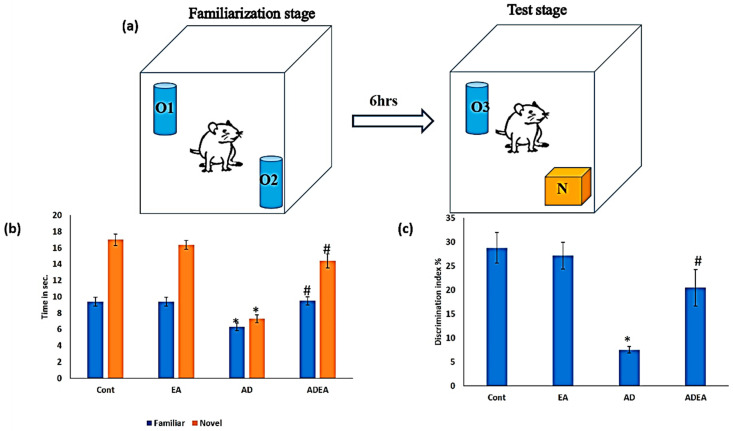
(**a**–**c**): Novel object recognition test. (**a**) A schematic diagram representing the stages of the NORT; (**b**) bar graph showing familiar and novel object exploration times spent by rats in all groups; (**c**) bar graph showing the DI between familiar and novel objects. Data are presented as the means ± SD. One-way ANOVA was used, and Fisher’s LSD *t*-test was applied when equal variance could be assumed. * Significantly different from the control, EA, and ADEA groups at *p* ≤ 0.05. # Significantly different from the AD group at *p* ≤ 0.05.

In the test stage, there were no significant differences in the exploration times for the old (O3) and novel (N) objects between the EA and control groups (*p* = 0.169 and 0.1, respectively). In the AD group, the exploration time for O3 decreased by 32% compared to the control and EA groups, while the exploration time for N decreased by 57 and 55.48% compared to the control and EA groups, respectively. ADEA rats explored N for a mean of 14.4 ± 0.84 s, which was an increase from 7.3 ± 0.48 s in AD rats. The DI increased significantly in the ADEA group compared to the AD group (*p* ≤ 0.05) (Figure 1a–c).

### 3.2. Antioxidant Biomarkers 

**Figure 2:**
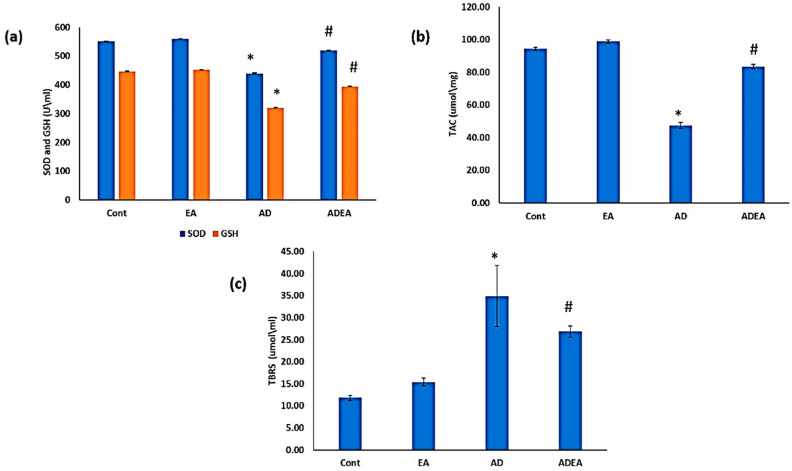
(**a**–**c**) Variations in antioxidant biomarkers and products of lipid peroxidation. (**a**) Bar graph showing significant decreases in serum SOD and GSH levels (U/mL) in the AD group, which were elevated upon treatment with EA; (**b**) bar graph showing variations in TAC with significant decrease in the AD group; (**c**) bar graph showing serum levels of lipid peroxidation products (TBRS). Data are presented as the means ± SD. One-way ANOVA was used, and Fisher’s least LSD *t*-test was applied when equal variance could be assumed. * Significantly different from the control, EA, and ADEA groups at *p* ≤ 0.05. # Significantly different from the AD group at *p* ≤ 0.05.

SOD and GSH: Serum levels of SOD and GSH decreased significantly in the AD group compared to the other groups (*p* ≤ 0.05). The ADEA group had significantly increased mean serum SOD (518.9 ± 1.59 U/mL) and GSH (395 ± 0.81 U/mL) levels compared to the AD group (SOD: 440.8 ± 2.25 U/mL; GSH: 321.1 ± 0.99 U/mL) (Figure 2a). The plasma TAC levels were significantly decreased in the AD group compared to the control and EA groups (*p* ≤ 0.05). The ADEA group had significantly increased mean TAC levels (83.6 ± 1.17) compared to the AD group (47.5 ± 1.84) Figure 2b). Levels of TBRS decreased significantly (*p* ≤ 0.05) following EA administration in AD rats, with mean values of 26.8 ± 1.25 μmol/mL and 34.9 ± 6.88 µmol/mL in the ADEA and AD groups, respectively (Figure 2c).

### 3.3. Histological Study

**Figure 3:**
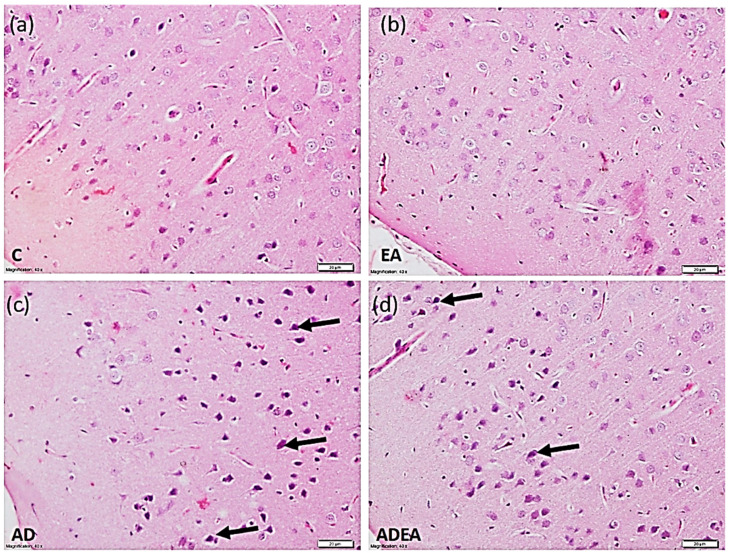
Photomicrograph showing the normal architectural pattern of the ERC with neurons having vesicular pale nuclei in the control (**a**) and EA (**b**) groups. In the AD (**c**) group, the ERC exhibited disturbed architecture, and neurons with condensed deeply stained pyknotic nuclei (arrows). In the ADEA (**d**) group, most neurons were restored with clear vesicular nuclei among a few scattered hyperchromatic condensed nuclei (arrows). (H&E, magnification ×40, scale bar 20 µm).

H&E-stained sections revealed normal structural pattern of the ERC in the control and EA groups, with pale vesicular nuclei in the neurons. In the AD group, the ERC exhibited a disturbed architecture and the neurons had condensed, deeply stained, pyknotic nuclei. In the ADEA group, most of the neurons had restored normal features among a few scattered hyperchromatic condensed nuclei (Figure 3).

**Figure 4:**
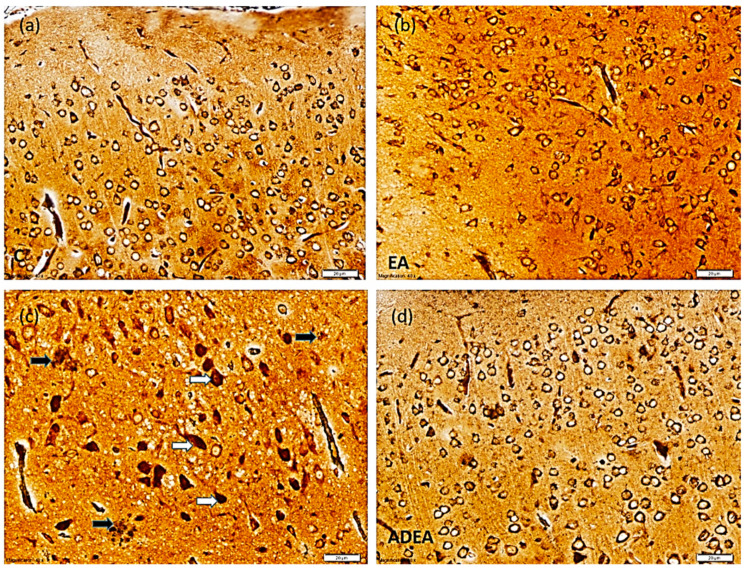
Photomicrograph showing normal ERC structure in the control (**a**) and EA (**b**) groups. The sections from the AD (**c**) group showed multiple NFTs (white arrows) with NPs in between (black arrows). Sections from the ADEA (**d**) group revealed a few scattered NFTs among normal neurons. (Silver, magnification ×40, scale bar 20 µm).

In the silver-stained sections, a normal ERC structure in the control and EA groups was evident. In the sections from the AD group, multiple NFTs with NPs were observed. EA administration in the ADEA group resulted in an apparent decrease in NFTs, which appeared scattered among the restored normal neurons (Figure 4).

### 3.4. ERC Thickness

**Figure 5:**
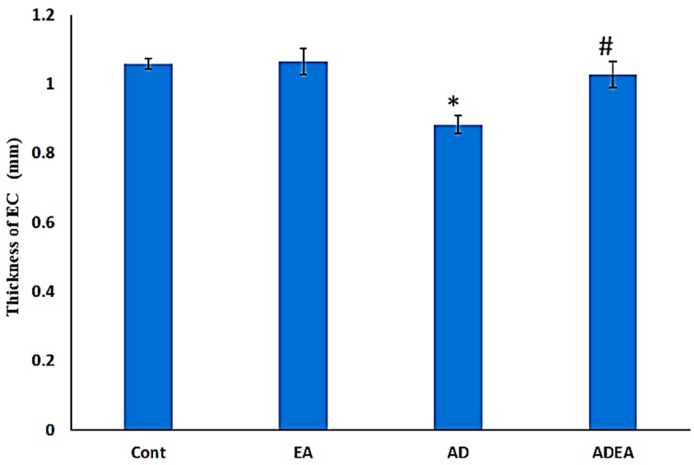
Bar graph showing the ERC thickness (mm) in the groups. Data are presented as the means ± SD. One-way ANOVA was used, and Fisher’s LSD *t*-test was applied when equal variance could be assumed. * Significantly different from the control, EA, and ADEA groups at *p* ≤ 0.05. # Significantly different from the AD group at *p* ≤ 0.05.

The AD group had a significantly decreased mean ERC thickness (0.83 ± 0.025 mm) compared to the control (1.06 ± 0.15 mm) and EA (1.06 ± 0.037 mm) groups. The ERC thickness in the ADEA group responded to EA therapy and increased by 22.89% (1.02 ± 0.37 mm) compared to the AD group (Figure 5).

### 3.5. Immunohistochemistry

**Figure 6:**
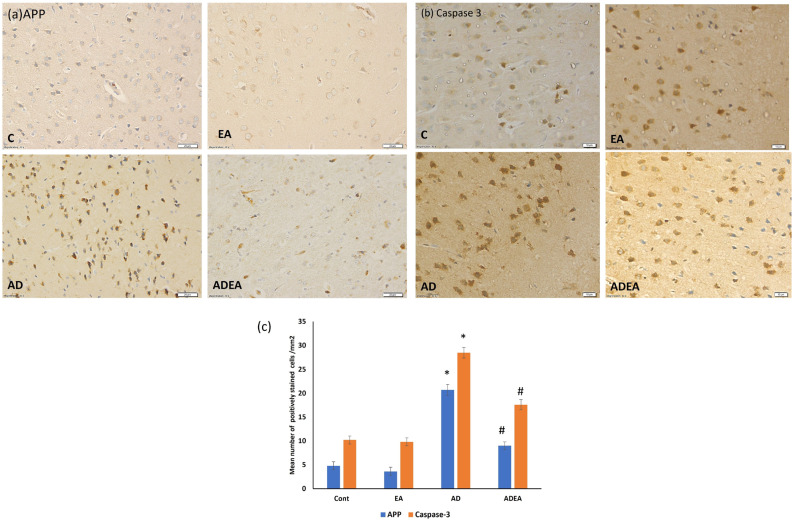
(**a**–**c**). Photomicrographs of ERC immunostained sections showing upregulated expression of (**a**) APP and (**b**) caspase-3 in the neurons from the AD group compared to the other groups. (Immunohistochemistry, magnification ×40, scale bar 20 µm.) (**c**) Quantitative analysis of the mean number of APP- and caspase-3-positively immunostained neurons per square millimeter. One-way ANOVA was used, and Fisher’s LSD *t*-test was applied when equal variance could be assumed. * Significantly different from the control, EA, and ADEA groups at *p* ≤ 0.05. # Significantly different from the AD group at *p* ≤ 0.05.

Immunostaining of ERC sections revealed that EA administration in the ADEA group significantly downregulated APP and caspase-3 expression (*p* ≤ 0.05) compared to the AD group. This was confirmed by the quantitative analysis of the mean number of APP- and caspase-3-positively immunostained cells per square millimeter (Figure 6a–c).

### 3.6. Changes in SOD mRNA Levels via q-PCR

**Figure 7:**
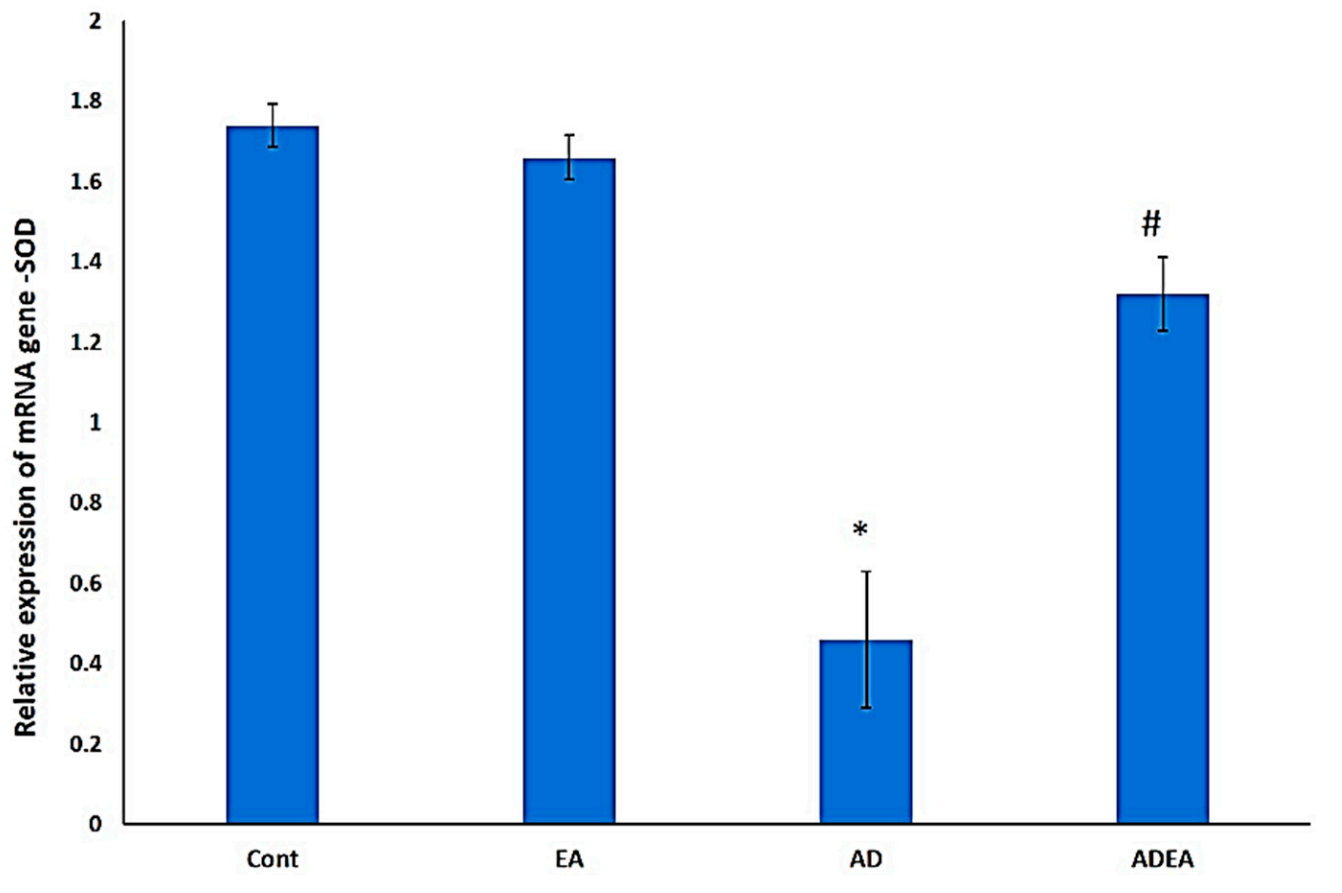
Bar graph showing the relative expression of *SOD* mRNA in ERC. One-way ANOVA was used, and Fisher’s LSD *t*-test was applied when equal variance could be assumed. * Significantly different from the control, EA, and ADEA groups at *p* ≤ 0.05. # Significantly different from the AD group at *p* ≤ 0.05.

*SOD* gene expression was significantly upregulated in ADEA rats (*p* ≤ 0.05) compared to AD rats. This suggested that EA administration mitigated oxidative stress by upregulating *SOD* expression (Figure 7).

## 4. Discussion

AD is a major cause of dementia. Although oxidative stress is a part of the normal aging process, it is also an early sign of AD [35]. Several human and experimental studies have reported ERC atrophy as the earliest sign of AD. A major pathological feature is Aβ aggregation in the outer ERC layers, which constitutes the major excitatory input to the hippocampus, and may eventually cause extensive neuronal death [36]. 

Behavioral tests, such as the Y-maze test, Morris water-maze test, and NORT, have been used to study memory [37,38,39]. In the present study, the NORT was used to study the effects of EA on episodic memory in a rat AD model. The results revealed a significant decrease in DI in AD rats, with no significant difference between the exploration times for old and novel objects. EA administration in AD rats increased the DI, as indicated by longer exploration times for the novel object compared to the familiar object. Similarly, other researchers reported a positive relationship between neurogenesis rate and NORT performance in several areas of the brain, including the dentate gyrus and ERC [40]. 

It is to be noted that biomarkers were evaluated in the present study due to their tight interconnection with AD hallmarks. SOD was chosen as Aβ was reported to inhibit mitochondrial superoxide dismutase (MnSOD), the enzyme most involved in the detoxification of the anion superoxide and protection from peroxidative damage [41]. Cu, Zn-SOD are fatally affected by the oxidative damage to the brain in AD and Parkinson’s disease [42]. In addition, GSH was proved to be low in brain tissue and blood in cases of AD and mild cognitive impairment [43], but higher plasma GSH levels were associated with a decreased risk of developing AD [44].

This finding was also correlated with the serum antioxidant profile, with significantly elevated SOD, GSH, and TAC in ADEA rats compared to untreated AD rats, which confirms that brain cells are highly sensitive to oxidative stress [45]. Elevated peripheral inflammatory markers and enhanced ROS generation result in the deterioration of cellular functions with a consequent degeneration of nervous tissue, which eventually leads to neurological and mental defects [46]. 

In the present study, the significant elevation of TBRS in the AD group was considered as evidence of the involvement of oxidative stress in the pathogenesis of AD, as reported previously [47,48]. These levels decreased significantly in ADEA rats. It was reported that plasma levels of oxidation protein products were increased in both mild cognitive impairment and AD [49]. Moreover, it has been suggested that lipid peroxidation may be involved in enzyme and signaling-protein malfunction through changes in the membrane milieu or through its products, such as reactive aldehydes, which are capable of engaging proteins [50]. 

Histopathological examination of the ERC revealed diminished thickness in the AD group compared to the ADEA group. Similar results were reported, where thicknesses of entorhinal and transentorhinal cortices in subjects with mild cognitive impairment were decreased by 0.6 mm compared to normal subjects [51]. In their experimental work on traumatic brain injuries, researchers observed chronic behavioral changes in mice concomitant with a decrease in the thickness of the contralateral ERC over a maximum period of 6.5 months, and attributed these to progressive brain degeneration [52]. The decrease in ERC thickness was also related to decreased TAC and elevated ROS; this can cause the destruction of proteins, DNA, and membrane fatty acids, and result in apoptosis, neurodegeneration, and volumetric changes in the brain [53]. Moreover, a decrease in ERC volume could predict the progression of early cognitive deterioration into AD [54]. 

Numerous characteristic NFTs and NPs of AD were identified in the silver-stained sections, while H&E staining demonstrated disturbed architecture and apoptotic neurons. Immunohistochemistry confirmed these results and quantitative analysis of APP and caspase-3-positive cells per square millimeter revealed that EA downregulated APP expression and decreased the number of apoptotic neurons.

Previously, the “amyloid stream” postulation has been used to explain that the formation of Aβ from APP is the first step in the pathological stream leading to toxic Aβ aggregation, decreased synapse plasticity, NFT formation, and eventually neuronal cell death [55,56]. Furthermore, ROS can trigger a lack of oxygen in the nervous tissue and Aβ toxicity, leading to neurodegeneration [57]. It has been reported that the amount and distribution of NFTs is directly related to the severity and duration of dementia [58]. 

Based on the initial strength and the period of pathologic exciter, neuronal death may be due to apoptosis or necrosis. While necrosis is a rapid process that cannot be terminated once started, apoptotic cell death can be delayed by the activation of neuroprotective and antiapoptotic mechanisms [59]. 

Caspase-3, an executioner caspase, is activated by initiator caspases and triggers the apoptotic cascade [60]. Caspase-3 can split APP and form a neurotoxic peptide (C31) that triggers the cytotoxic Aβ, leaving Tau in the C-terminal region, and causes NFT formation [61]. This results in the failure of synaptic suppleness and normal learning activity. Therefore, the neuronal cell death in AD was attributed to apoptosis and its synergistic action with ROS [62]. 

In the present study, EA administration enhanced *SOD* gene expression, which markedly diminished APP and caspase-3 expression, and consequently attenuated the pathological features of AD in ERC tissue. Other studies have reported that increased *SOD* gene expression reduces lipid peroxidation [63] and plaque formation, leading to decreased memory deterioration [64]. Principally, SOD supplementation showed improvement in mice model of AD in a previous experiment [65]. It was also reported that EA not only eliminated superoxide and hydroxy anions, but its effect was stronger than that of α-tocopherol and it was as potent as SOD [66]. Other researchers concluded that the neuroprotective effect of EA against methyl-4-phenyl 1,2,3,6 tetrahydropyridine (MPTP) neurotoxicity was through inhibiting oxidative stress, increasing the antioxidant enzymes/peptide, and preventing the activation of inflammatory cytokines and their mediators [67]. Moreover, the effect of EA on a parkinsonism rat model was evaluated and it was revealed that EA can improve the disturbed motor function and increase the cerebral antioxidant defense [68].

## 5. Conclusions

In conclusion, besides the antioxidant properties of EA, it also contributed to SOD gene modulation, changes in the ERC antioxidant milieu, and a reduction in the oxidative stress, which mitigated APP toxicity and caspase-3-mediated apoptosis. Consequently, it restored episodic memory and serum antioxidant biomarkers, and curtailed the histopathological AD hallmarks, such as NFTs and NPs, in an AD rat model. Ellagic acid proved to be a powerful modulator of oxidative stress by enhancing antioxidant biomarkers in serum and SOD gene expression in the brain, thus it can be considered a promising therapeutic measure for AD.

## Figures and Tables

**Table 1:** Primer sequences for GAPDH and SOD genes.

Gene Expressed	mRNA Primer Sequence
SOD1	Forward: 5′AATGTGTCCATTGAAGATCGTGTGA3′ Reverse: 5′GCTTCCAGCATTTCCAGTCTTTGTA3′
GAPDH (internal control)	Forward: 5′GCACCGTCAAGGCTGAGAAC3′ Reverse: 5′ATGGTGGTGAAGACGCCAGT3′

## Data Availability

The datasets used and/or analyzed during the current study are available from the corresponding author on reasonable request.
