# Correction: Ramadan, W.S.; Alkarim, S. Ellagic Acid Modulates the Amyloid Precursor Protein Gene via Superoxide Dismutase Regulation in the Entorhinal Cortex in an Experimental Alzheimer’s Model. *Cells* 2021, *10*, 3511

**DOI:** 10.3390/cells15020108

**Published:** 2026-01-08

**Authors:** Wafaa S. Ramadan, Saleh Alkarim

**Affiliations:** 1 Department of Anatomy, Faculty of Medicine, King Abdulaziz University, Jeddah 21589, Saudi Arabia; 2 Department of Biological Sciences, Faculty of Science, King Abdulaziz University, Jeddah 21589, Saudi Arabia; skarim@kau.edu.sa

In the original publication [1], there was a mistake in Section 2, the Institutional Review Board Statement, and Figure 6 as published.

In Section 2. Materials and Methods, in the first paragraph and second phrase, the “Declaration of Helsinki” was mentioned as one of the principles applied to this article. As this study did not involve any humans, the statement was removed, as was reference 26. The remaining references were rearranged.

The Institutional Review Board Statement was updated, and reference to the “Declaration of Helsinki” was removed.

In Figure 6b, due to an error in the panel arrangement, there was an image overlap between panels C and EA. The updated Figure 6 appears below:

**Figure 6:**
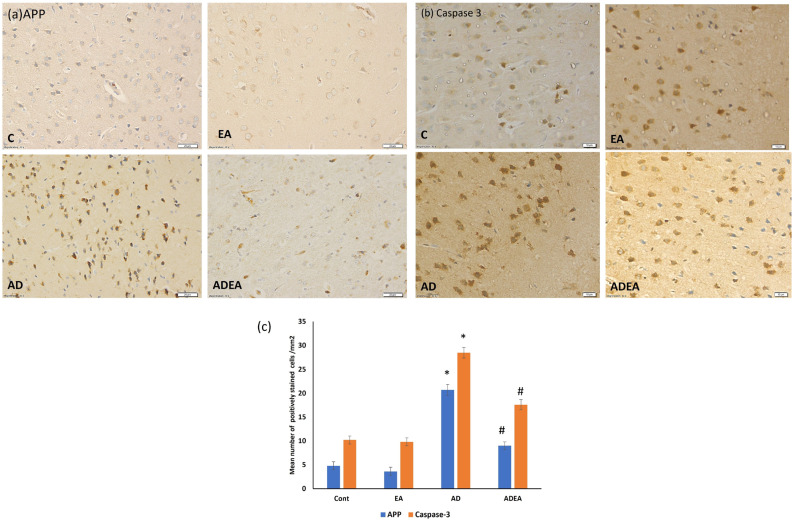
(**a**–**c**). Photomicrographs of ERC immunostained sections showing upregulated expression of (**a**) APP and (**b**) caspase-3 in the neurons from the AD group compared to the other groups. (Immunohistochemistry, magnification ×40, scale bar 20 µm.) (**c**) Quantitative analysis of the mean number of APP- and caspase-3-positively immunostained neurons per square millimeter. One-way ANOVA was used, and Fisher’s LSD *t*-test was applied when equal variance could be assumed. * Significantly different from the control, EA, and ADEA groups at *p* ≤ 0.05. # Significantly different from the AD group at *p* ≤ 0.05.

The authors state that the scientific conclusions are unaffected. This correction was approved by the Academic Editor. The original publication has also been updated.
